# Priming, Triggering, Adaptation and Senescence (PTAS): A Hypothesis for a Common Damage Mechanism of Steatohepatitis

**DOI:** 10.3390/ijms222212545

**Published:** 2021-11-21

**Authors:** Peter M. Abuja, Kurt Zatloukal, Helmut Denk

**Affiliations:** Diagnostic & Research Center for Molecular Biomedicine, Diagnostic & Research Institute of Pathology, Medical University of Graz, A-8010 Graz, Austria; kurt.zatloukal@medunigraz.at (K.Z.); helmut.denk@medunigraz.at (H.D.)

**Keywords:** steatohepatitis, pathomechanism, metabolism, stress defence, mitochondrial damage, hypoxic signalling, senescence, cirrhosis, hepatocellular carcinoma

## Abstract

Understanding the pathomechanism of steatohepatitis (SH) is hampered by the difficulty of distinguishing between causes and consequences, by the broad spectrum of aetiologies that can produce the phenotype, and by the long time-span during which SH develops, often without clinical symptoms. We propose that SH develops in four phases with transitions: (i) priming lowers stress defence; (ii) triggering leads to acute damage; (iii) adaptation, possibly associated with cellular senescence, mitigates tissue damage, leads to the phenotype, and preserves liver function at a lower level; (iv) finally, senescence prevents neoplastic transformation but favours fibrosis (cirrhosis) and inflammation and further reduction in liver function. Escape from senescence eventually leads to hepatocellular carcinoma. This hypothesis for a pathomechanism of SH is supported by clinical and experimental observations. It allows organizing the various findings to uncover remaining gaps in our knowledge and, finally, to provide possible diagnostic and intervention strategies for each stage of SH development.

## 1. Introduction

### 1.1. What Is Steatohepatitis?

Fatty liver disease is a multifactorial health problem with world-wide increasing prevalence [1,2,3,4]. It is the result of a complex interplay between the liver, adipose tissue, and intestine. According to the aetiology, fatty liver disease is categorized as alcoholic or non-alcoholic. The latter (i.e., non-alcoholic fatty liver disease; NAFLD) arises in the absence of significant alcohol consumption and is the hepatic manifestation of the metabolic syndrome, characterized by visceral obesity, dyslipidaemia, type II diabetes mellitus, insulin resistance (IR), elevated arterial blood pressure, and related cardio-vascular disorders [3,5,6,7,8]. Both disorders may progress from simple steatosis to the necro-inflammatory lesions of alcoholic (ASH) and non-alcoholic (NASH) steatohepatitis (SH), respectively, with cirrhosis and hepatocellular carcinoma as late-onset sequelae [2,3,9].

Fatty liver disease in humans is variable in its morphologic and clinical appearance, depending on the aetiology, severity, and stage [10,11]. It may be caused by a broad spectrum of insults, including alcohol abuse and metabolic disorders related to an imbalance of nutrient intake, energy expenditure, and acute or chronic drug intoxication (drug-induced liver injury, DILI) [12,13,14,15]. Its severity ranges from the relatively benign and reversible fatty liver (termed simple steatosis) to steatohepatitis (SH). SH is characterised by inflammation, hepatocellular damage with necrosis, apoptosis, ballooning, derangement or loss of the keratin intermediate filament cytoskeleton and formation of protein aggregates termed Mallory–Denk bodies (MDBs) [5,16,17], altered energy metabolism [18], oxidative stress, as well as damage to and deficient quality control of mitochondria [19,20,21,22,23,24,25]). It can eventually progress to cirrhosis and hepatocellular carcinoma [2,3,9,26].

### 1.2. Current State of Knowledge about the Molecular Disease Mechanism

Human SH is characterised by alterations that include morphological changes as key diagnostic criteria [5,16,17], metabolic disturbances [18], oxidative stress [19,23,27,28,29], and inflammation. In addition, it is justified to add cirrhosis and hepatocellular carcinoma (HCC) [9,26] to the phenotypical characteristics of the disease.

A remarkable feature of SH is that it can be the result of a broad aetiological spectrum that nevertheless converges to the same phenotype.

A vast body of literature has accrued over the past decades reporting numerous details regarding contributors to SH, including but not limited to metabolic alterations involving energy metabolism [18], oxidative damage [19,23,27,28,29], mitochondrial dysfunction [19,23,25,27,30], hypoxic response [31,32,33,34,35], and senescence [36,37,38,39], as well as the roles of adipose tissue [40], proinflammatory cytokines [41], and the microbiome [42,43,44]. However, there is presently only limited knowledge about the pathogenic mechanism responsible for the severe forms, particularly the necro-inflammatory lesions in ASH and NASH, respectively. To some extent this is due to the fact that these chronic diseases remain clinically silent for a rather long time (1–5 years for ASH, even longer for NASH, where the onset of insult is often undefined [45]). Moreover, no reliable non-invasive screening procedures are available—the gold standard for the diagnosis of SH is still histopathological examination of liver biopsy. Hence, ASH and NASH are usually detected at a late stage of their development, which explains the insufficient knowledge about their early stages and molecular triggers. Furthermore, while allowing histological assessment, formalin-fixed biopsy material precludes obtaining sufficiently detailed functional data, e.g., about causes and consequences of molecular alterations and mitochondrial function. Animal models have supported the mechanistic research in this respect (see also below) [23,24,25,46] but often give only circumstantial evidence for human SH [16].

### 1.3. A Mechanistic Framework Is Urgently Needed

Although a considerable amount of data has accumulated over the past decades, the available information has not yet been assembled into a consistent molecular framework that covers all the stages of SH development, including sequelae. Because the processes are highly interconnected, it is difficult to distinguish between causes and consequences, which is, however, indispensable to understand the pathogenesis and to discover and eventually fill gaps in our understanding.

In a complex disease such as NASH, understanding the pathogenesis requires knowledge of the evolution of the disease process from initial damage, via the ‘mature’ phenotype, to sequelae. This is indispensable for the design of screening methods and of efficient therapeutic or preventive strategies. Many of the alterations described in the literature are not drivers but rather consequences of underlying, primary molecular events that affect metabolism, stress response, as well as intra- and intercellular signalling. Identifying drivers, in their chronological sequence, is therefore a useful strategy for the elucidation of the causal chain finally responsible for the fully developed disease.

The evidence for our proposed mechanism of SH development presented below often resulted from human and animal studies without direct reference to SH. However, these studies can explain basic underlying principles that should be confirmed (or refuted) in the context of SH. Moreover, some aspects of the model we present here are substantially underpinned by experimental findings, while other parts, specifically the later phases, are more hypothetical and will need specific research.

### 1.4. How Can a Mechanistic Framework Be Found?

The working strategy is to uncover events that causally link the primary damage (i.e., immediately resulting from the primary insult) to the final (predominantly morphologic) phenotype, including potential progression to sequelae. This approach allows distinguishing those events that eventually lead to the phenotype from other alterations that occur along the transition from normal to diseased liver. As usual in natural sciences, the mechanism that we describe here is not the only possible one, but it is consistent with a causal chain that is supported by a large body of findings.

## 2. A Proposed Common Damage Mechanism (CDM) of SH: Priming/Sensitizing, Triggering, Adaptation and Senescence/Sequelae (PTAS)

It is striking that different aetiologies can lead to the SH phenotype. Since each aetiology initially produces a specific type of insult it is likely that the different primary insults converge on a common damage mechanism (CDM). As shown below, the initial damage by the different aetiologies resembles the well-known hypotheses about two or more ‘hits’ [47,48]. Convergence on a CDM can occur early in disease development if the different aetiologies produce similar insults. Alternatively, different primary insults may converge late on a CDM; in this case it may be a secondary mechanism that leads to the SH phenotype which is not a direct consequence of the primary damage.

We favour the second option because the mature phenotype of NASH takes a long time to develop after the initial damage. It was claimed that simple steatosis may be a precursor state for NASH [11,26,49]. We will show in the next section how steatosis fits into a mechanistic model as an initial cause. Moreover, from our previously published work [28,50] and the work of others, we deduce that the development of the SH phenotype requires at least two sequential or parallel insults to hepatocytes prior to the CDM. In more general terms this was already proposed as the ‘two (or multiple)-hit hypothesis’ [47,48], however without the addition of a CDM. Furthermore, from observations in humans and experimental animals we consider the CDM to involve metabolic adaptations following the initial, acute insults that allow tolerating the damage and maintaining basic liver function, in the sense of a chronic disease. Adaptation appears to result in cellular senescence and thus at least temporarily prevents neoplastic transformation that might otherwise be triggered by adaptive metabolic alterations. We propose that adaptation is the committed step of the SH phenotype development; it constitutes a metabolic state of the liver that is essentially functional, but different from normal metabolism, and may be responsible for inflammation through the senescence-associated secretory phenotype (SASP).

It is likely that the altered metabolic state reached during adaptation persists even when the morphological phenotype is resolved after some time of absence of the aetiologic cause. This is observed in ASH patients upon ethanol withdrawal, or in certain toxicity-induced mouse models [51], when the phenotype-inducing toxin is withdrawn; upon re-intoxication, the morphological phenotype will reappear rapidly—actually much more rapidly than upon first-time intoxication. This indicates that the CDM induces an altered metabolic state (the secondary damage mechanism mentioned above), which is responsible for the complex morphological phenotype and the well-known sequelae. Of note, this concept allows that the altered metabolic state will provide susceptibility to any further insult that can induce acute damage.

We propose a four-stage mechanism for SH: (i) a priming event that by itself induces no, or only minor, hepatocellular damage but impairs stress defence capacity and thus sensitizes the cell to further insults; (ii) a trigger that induces the actual (or acute) damage, which is exacerbated by the diminished defence capacity. We posit that in absence of prior priming, the trigger alone can induce some damage, which is, however, mitigated by the defence and repair capacity of the cell. Finally, (iii) irreversible adaptation of the metabolic state initiates a CDM that preserves basal hepatocellular and organ function under sustained conditions of priming and triggering, develops the characteristics of the SH phenotype, and (iv) ends up in cellular senescence that, after escape from this state, favours development of hepatocellular carcinoma (HCC).

## 3. Experimental Evidence Supporting PTAS

### 3.1. Priming Leads to Compromised Stress Defence

Experimental evidence for the existence of the first two stages, namely priming and triggering, has been obtained with a mouse model based on prolonged dietary administration of the porphyrinogenic substance 3,5-diethoxycarbonyl-1,4-dihydrocollidine (DDC; 0.1% (*wt/wt*) for 8–10 weeks) leading to inhibition of ferrochelatase [52,53]. In this model, acute hepatocellular damage with focal apoptotic and necrotic cell death occurs early (during the first 2–5 weeks of DDC administration), whereas development of the SH phenotype requires 8–10 weeks of continuous DDC treatment [50,51]. A similar situation was observed in ferrochelatase-deficient mice [29]. In the acute intoxication stage, DDC primes the liver by suppression of the Nrf2-dependent stress response [50]. In this context, a significant downregulation of *Pparα* and PPARα-controlled genes [50] occurred, affecting not only genes involved in energy and lipid metabolism but also typical stress-response genes, such as *Gclc*, *Sod1*, *Sod2,* and *Hmox1*. The effects of priming persisted for several months after cessation of DDC administration (P.M.A., et al., unpublished data), probably as a result of an irreversible metabolic alteration due to the interplay of PPARα-mediated lowering of stress response [50] in combination with c-Myc [54,55], succinate efflux from hepatocytes [56] leading to persistent hypoxic signalling [57,58]. This may explain the rapid reappearance of the SH phenotype during re-intoxication [59]. The trigger may be based on the dysfunction of mitochondrial complex II [28], probably caused by inhibition of haem production and resulting in impaired formation of haem-containing proteins. Table 1 summarizes the priming factors.

Of note, downregulation of *Ppara* and PPARα-dependent genes by itself does not lead to damage and does not (yet) produce the SH phenotype but only lowers resilience towards stress. That is the reason why *Ppara*-deficient mice do not spontaneously develop SH unless there is an additional trigger, such as ethanol [60].

**Table 1:** Factors that prime the liver for extensive damage after triggering.

Factor	Mechanism	Model
Porphyrinogens (DDC, griseofulvin); liver toxins	AhR ↑ → PPARα ↓ & c-Myc ↓ → Nrf2-dependent genes ↓	Intoxication mouse models [16,28,50,51,52,53,61,62]; DILI [12,13,14]
High-fat diet ^1^	AhR ↑ → PPARα ↓ & c-Myc ↓ → Nrf2-dependent genes ↓	HFD mouse model; human NASH [16,63,64,65,66,67,68]
High-fat diet ^1^	Palmitoyl-CoA → NNT inhibition → NADPH_mito_ ↓ → GSH_mito_	HFD mouse model; human NASH [69,70,71,72,73]
Excess keratin 8	Impaired mitochondrial QC via Pirh2	Keratin 18^−/−^ mouse model, human NASH [16,59,74,75,76,77,78,79,80,81]

^1^ HFD in normal experiments does not lead to the SH phenotype [16], most likely because HFD only primes but needs a separate trigger.

#### 3.1.1. Mechanistic Implications of PPARα Downregulation

PPARα is predominantly regarded as a regulator of energy (lipid) metabolism in the liver. However, PPARα also activates the Nrf2-dependent stress response [50], very likely via c-Myc [54,55]. It is connected with hypoxic signalling and mitochondrial QC [57,82,83].

The initial downregulation of PPARα may be caused by activation of the aryl hydrocarbon receptor [67] by oxidative stress [84], or (e.g., in the DDC model) by porphyrin degradation products, such as bilirubin [68]; this notion is corroborated by observations in ferrochelatase-deficient mice that also develop porphyria and the SH phenotype [29]. Another possibility is that oxidative stress directly leads to stabilization of hypoxia-inducible factors [57,85], which then contribute to persistent PPARα downregulation at later stages of the disease. A similar pattern of downregulation of stress response genes was described for mice fed a high-fat diet (HFD) [86], although these mice did not develop the SH phenotype after prolonged HFD feeding. This may be due to a lack of a suitable trigger, which could be, e.g., an imbalance of the keratin 8/18 content of the cell [65,74,75,79] (see below), or due to insufficient time for phenotype development. Similarly, it was shown recently that hepatocyte-specific ablation of PPARα induces steatosis, but not SH [66].

Cross-talk between PPARα and the Nrf2-controlled stress response has been shown to affect the expression of a wide variety of genes [87,88,89]. Since suppression of stress response also affects the redox state of the hepatocyte and its mitochondria, in particular the glutathione (GSH) redox buffer, this is probably a key element of the reduced resilience to subsequent insults, revealed by impaired mitochondrial function and increased oxidative damage [28].

Recent studies showed that expression of PPARα target genes may be amplified by c-Myc [90], which enables the full stress response via Nrf2 [54,55]. Conversely, downregulation of PPARα reduces this amplification.

Another factor that determines stress response is the mitochondrial NADPH pool, which is required to keep the sulfhydryl cofactors, specifically GSH, in a reduced state. Mitochondrial NADPH is regenerated from NADP^+^ in the tricarboxylic acid (TCA) cycle and, also, with NADH as the electron donor, through nicotinamide nucleotide transhydrogenase (NNT), a proton-translocating enzyme located in the inner mitochondrial membrane [70,91]. In addition to the well-known genetic NNT defect in the C57BL/6J mouse substrain [72,73,92,93] that severely affects their mitochondrial stress-resilience, the function of NNT may be compromised by a variety of biogenic inhibitors [69]; one of them is palmitoyl-CoA, which may be relevant in HFD-fed animals and human steatosis [94].

#### 3.1.2. Priming in Human SH

Since priming occurs early in the development of SH in humans, persistent markers are likely to serve as indicators. The persistent downregulation of PPARα found in mice [50] is also found in patients with SH [61] but not in patients with simple steatosis [31,62,95]. Agonists for PPARα and other isotypes showed promising therapeutic effects [96,97] similar to the observations in mice, A recent review and meta-analysis of the efficacy of the PPARα/δ agonist elafibranor in clinical trials reported that it improved most metabolic parameters, particularly serum lipid profiles and liver enzymes [98].

#### 3.1.3. Priming Is a Factor Determining Sexual Dimorphism

Recently it was shown that the downregulation of PPARα exhibits pronounced sexual dimorphism in diet-induced NAFLD in mice, as female mice were protected from the adverse effects of a hypercaloric diet [99]. A similar sexual dimorphism of PPARα and PPARα-controlled genes has been shown in humans with simple steatosis, moderate fibrosis, and absence of ballooning and, therefore, did not relate to SH [99].

### 3.2. Triggering Induces Severe Hepatocellular Injury

The factors that prime and trigger may differ with regard to aetiology. In many cases, the triggering event cannot be clearly separated from priming. For example, in the DDC-intoxication mouse model the induction of porphyria may be responsible both for priming, by lowering PPARα expression, as well as for triggering, since the production of ferrohaem is inhibited leading to mitochondrial dysfunction. This is most likely due to impaired synthesis of haem-proteins of the electron transport chain, resulting in impairment of the tricarboxylic acid cycle and increased formation of reactive oxygen species (ROS) [28,100,101,102]. Particularly prominent was the inhibition of succinate:quinone oxidoreductase (SQR; also termed succinate dehydrogenase, SDH; or complex II) in the DDC mouse model within about two weeks [28,50], accompanied by substantial weight loss and 40% lower hepatic ATP content. Consequences of mitochondrial dysfunction, such as reduced ATP content, were also observed in human ASH [103] and NASH [22,30]. Another cause of SQR inhibition may be superoxide production by mitochondrial respiratory complex I [104]. Succinate produced in the TCA cycle is oxidized by SQR with concomitant reduction of ubiquinone to ubiquinol in the mitochondrial electron transport chain (TCA). Besides contributing directly to oxidative damage by production of ROS [71,102,105], SQR dysfunction can impair both ETC and TCA cycles, leading to increased succinate and succinyl-CoA concentrations. Succinyl-CoA is an inhibitor of the upstream TCA cycle enzymes, α-ketoglutarate dehydrogenase and citrate synthase, which is the likely cause of the reduced ATP synthesis after inhibition of SQR activity [28]. On the other hand, succinate induces hypoxic signalling by stabilizing hypoxia inducible factor 1α (HIF-1α) and Hif-2α through (product) inhibition of the HIFα prolyl hydroxylases (PHD) [34].

In line with observations in other models and in human SH, elevated succinate is a signal of liver damage. It can bind to a G-protein-coupled succinate receptor, SUCNR1 [106], that activates hepatic stellate cells [107,108,109], triggers an immune response [56,110,111], and impairs the mitochondrial quality control machinery [112]. Moreover, it has been demonstrated in an acetaminophen-toxicity model that, by uncoupling the succinate dehydrogenase and quinone reductase activities of SQR with methylene blue, the adverse effects of SQR inhibition can be relieved [113].

Interestingly, it was recently described that uncoupling protein 1 (UCP1) in adipocytes [40], which is also expressed in the kidney [114] and in hepatocytes [115], may influence the systemic and hepatic succinate pools [40], which is a possible link between obesity and liver disease.

The impairment of the TCA cycle eventually lowers formation of NADPH, required for the regeneration of (mitochondrial) glutathione (GSH). Together with the unbalanced utilization of TCA cycle substrates, which favours ROS formation, the reduced antioxidant capacity of the GSH pool may promote damage of key enzymes of the TCA cycle and ETC.

#### 3.2.1. Triggering in Human SH

Similar to some animal models, the identification of a triggering insult in human SH is hampered by the difficulty to choose the right time, i.e., before adaptive processes start; early events in the development of SH can usually not be detected in isolation. However, several consequences persist for a longer time period, such as lower hepatic ATP levels [30,116], succinate efflux [101,106], and inflammatory processes [56], accompanied by oxidative and mitochondrial damage. Damage is not a result of priming alone, and it persists during adaptation to some extent since it cannot be completely mitigated, e.g., in NASH patients, the activities of all respiratory electron transfer complexes were found reduced, most pronouncedly complex II [20,22,71]. Table 2 summarizes triggers for mitochondrial damage and Table 3 the outcome of both priming and triggering.

#### 3.2.2. Triggering by Mitochondrial Dysfunction May Involve the Intermediate Filament Cytoskeleton

Mice fed a high-fat diet (HFD) show reduced expression of PPARα [63,64,86,118,119], but normally do not produce the SH phenotype, probably due to lack of a suitable trigger or too short duration of treatment. Mitochondrial dysfunction, prominent in human SH [19,27,120], was identified as the triggering event in the DDC-model as a consequence of haem deficiency, leading predominantly to inhibited function of SQR [28]. A first indication of how cytoskeletal elements could lead to mitochondrial dysfunction came from early experiments where griseofulvin-treated mice recovered for one month on a normal diet, upon which the SH phenotype disappeared. Re-challenge of these mice with either griseofulvin or colchicine (but not lumicolchicine or cytochalasin B) led to rapid reappearance of the SH phenotype [121]. In contrast to DDC, both griseofulvin and colchicine [122] interact with the tubulin cytoskeleton, and microtubules were described to be required for MDB formation [123]. Re-challenging DDC- or griseofulvin-fed mice after recovery with DDC achieves the same effect, and an excess of keratin 8 over keratin 18 was required to precipitate the SH phenotype [51,59,75,81,124,125].

The implication of keratin 8 (or the ratio keratin 8/18) as a trigger was corroborated by the finding that HFD-fed mice overexpressing keratin 8 developed the SH phenotype [65], which may be related to their propensity to be deposited as β-pleated sheet aggregates under these conditions [124]. Moreover, keratin 18-deficient mice (which only express keratin 8) spontaneously develop the SH phenotype (MDBs containing only keratin 8 are formed) [79]. Aggregation and deposition of misfolded proteins do probably not contribute to a CDM [125]. However, it is difficult to assess whether they are causally involved or merely represent a marker of the underlying processes. Increased levels of misfolded protein oligomers are known to be the toxic principle in protein misfolding diseases; therefore, they may contribute to maintaining the disease process, e.g., by sustaining mitochondrial dysfunction and ROS production (reviewed in [125,126]).

It is as yet unclear how the imbalance of keratin 8 and 18 triggers mitochondrial dysfunction; however, one possibility is the disturbance of intracellular mitochondrial distribution after the disruption of the interaction of the E3 ubiquitin ligase Pirh2 with the keratin intermediate filament, a process that may alter mitochondrial quality control [74]. Since this interaction is affected by phosphorylation of either Pirh2 [74] or the keratins, this may explain why the genetic background (in mice and humans) [75,78,80] plays an important role for the susceptibility for developing the SH phenotype. Pirh2 regulates stability and degradation of p53 and c-Myc [76] and could, thus, contribute to the persistent downregulation of the stress response [55,90] and to persistent mitochondrial damage [127,128,129].

Interestingly, Pirh2 also targets HuR [130], which is part of an RNA binding protein network that is activated during the HIF-2α-mediated hypoxic response [131]. HuR was reported to activate p62/SQSTM1-dependent selective autophagy in several cell types [132,133,134] and prevent steatosis in high-fat diet fed mice [135], possibly by increasing the stability of *Pten* mRNA. Moreover, HuR is also centrally involved in cell cycle regulation and might therefore link the immediate damage to adaptation and senescence [77]. This hypothesis might be tested, e.g., by using the keratin 18-deficient mouse model for SH.

### 3.3. Adaptation—Mitigation of Acute Hepatocyte Damage and Involvement of Other Cell Types

Adaptation, leading to the SH phenotype and probably transitioning to senescence, is the most complex part of the PTAS model since several different processes are deployed both in parallel and in succession: the hypoxic response, mediated by Hif-1 and Hif-2, the wound-healing response, inflammation, autophagy and mitophagy, among others. Therefore, different aspects of this phase may be observable at different time-points, and may partially obscure each other, in particular in human SH, but also in the better controlled environment of animal models. Therefore, more evidence specifically for this phase is needed for better understanding.

As indicated above, mitochondrial damage leads to impairment of the capacity of the TCA cycle to produce ATP, probably due to defective SDH, and succinate efflux into the cytosol. Elevated levels of succinate were described as a paracrine liver damage signal [106] and elicit a (pseudo)hypoxic response, similar to ischemia [136], by relieving the inhibition of Hif-1/2α through product inhibition of PHD. It allows survival of the hepatocyte but is also proto-oncogenic: succinate links metabolic deregulation to initiation of carcinogenesis, by transformation of cell function [137] and suppression of DNA repair [138]. These events, in addition to oxidative stress, trigger the development of a senescent state, especially the DNA-damage response (DDR) that is known to induce cell-cycle arrest, possibly as a means to prevent neoplastic hyperproliferation. Moreover, besides the activation of stellate cells [101] and triggering neoplastic transformation [139,140], succinate is involved in cross-talk of adipocytes with hepatocytes [40] and activation of immune cells [56,110].

In fibroblasts, both Hif-1 and Hif-2 induction leads to cell cycle arrest, without hypoxia, which may also represent senescence [141]. Interestingly, while the activation of hypoxia-dependent gene products occurs via Hif-1α, Hif-2α seems to involve translational modulation that increases formation of specific hypoxia-related proteins [142]. It was found that Hif-2α initially leads to a shutdown of transcription, while chronic hypoxia induces the Hif-2α-mediated remodelling of the central carbon metabolism by activating a specific translation initiation factor, eIF5B [143]; this process may also be operative during the chronic pseudohypoxic state described above. Recently, a network of RNA-binding proteins activated by Hif-2α was described [131], one of which, HuR, appears to be a target of the E3 ubiquitin ligase Pirh2 [130], which is involved in mediating mitochondrial localization [74]. This constitutes another link between hypoxia and cell cycle arrest as a consequence of the DDR [77].

Of note, Hif-2α was also described to activate the mTORC1 complex and to override the inhibition by Hif-1α in the liver and lung [144]. Interestingly, in peripheral blood mononuclear cells, upregulated expression of hypoxia-inducible genes was found in patients with chronic liver disease [145]. Hypoxic signalling might also be triggered focally by reduced hepatic microcirculation [145,146].

#### Mitigation of Damage and Restoration of Stress Response

Mouse models have shown that the initially suppressed stress response is restored [28], and typical stress proteins, such as haem oxygenase 1 or SQSTM1/p62 [51], are upregulated. Upregulation of SQSTM1/p62 is an important factor in the formation of MDBs [59,125,147,148,149] and is involved in autophagy and stress defence [150,151,152,153]. SQSTM1/p62 plays a role in the activation of stress response, by inducing selective autophagy and mitophagy; both may be independent [154] or dependent on a PINK1/PARKIN-related mechanism [155,156]. Mitophagy is apparently enhanced by ULK1 [155,157,158,159,160] that enhances binding to PINK1. On the other hand, SQSTM1/p62 directly activates the Nrf2-dependent stress response by interacting with Keap1, leading to its proteasomal degradation [153,161,162].

Moreover, ULK1 (re-)activates the stress response by enhancing the binding of SQSTM1/p62 to KEAP1 [157] followed by its autophagic clearance [162,163] and activation of the Nrf2-dependent induction of stress response proteins [164,165]. Interestingly, however, a splice-variant of SQSTM1/p62 lacking the Keap1-interacting region suppressed the stress response by increasing the amount of Keap1 [166].

Adaptation appears also to involve the activation of developmental pathways that are primarily responding to liver injury (excellently reviewed in [167]), such as Notch [168], possibly induced in stellate cells by osteopontin released from hedgehog-activated hepatocytes [169] as a response to injury [170].

Although sirtuins (Sirts) are probably not drivers of the CDM, they may play an important role in linking priming and triggering to the adaptive process and ageing. Therefore, and since the implications of NAD^+^ metabolism and Sirts with the CDM is beyond the scope of this paper, we provide only a few examples for this link; the dependence of Sirts on NAD^+^ implicates energy metabolism [171]. The deacetylase and desuccinylase activities, specifically of Sirt3 (acting exclusively on mitochondrial proteins), regulate mitochondrial function and the stress response [172]. Activation of the aryl hydrocarbon receptor (e.g., by oxidized fatty acids) deactivates Sirt3, which in turn lowers SOD2 activity and contributes to reduced mitochondrial stress defence [173]. This also results in altered energy homeostasis, which eventually favours sequelae such as HCC [172]. Sirt1 [174] and Sirt7 [175] have been implicated in the regulation of the hypoxic response via Hif-1 and thus constitute a potential link to ageing: Sirt1 is downregulated by autophagy in senescence [176], and there are links between overexpression of the cell-cycle regulator p16^INK4a^, Sirt1, and PPARα.

### 3.4. Senescence Prevents Neoplastic Transformation

Cellular senescence is a known tumour suppressor mechanism. Chronic liver disease, specifically SH, is often accompanied by senescence [39], in particular of hepatocytes [177,178,179,180,181] and cholangiocytes [182,183]. There is strong indication that many of the determinants for adaptation eventually culminate in senescence: mitochondrial dysfunction [100,184,185,186,187], metabolic alterations such as hypoxic signalling [129], oxidative stress [91,188,189], or downregulation of PPARα [190]. Importantly, there are potential routes to some of the hallmarks of the SH phenotype involving senescence, such as the formation of Mallory–Denk bodies [191].

### 3.5. Sequelae of SH—Cirrhosis and HCC

Hypoxic signalling has been found to be associated with hedgehog signalling, at least in paediatric NASH [35]. The hedgehog pathway plays a role in wound-healing and the response of the liver to injury [170]. Aberrant activation of hedgehog signalling was reported to be involved in development of HCC [192] by inducing metabolic changes in myofibroblasts that provide lactate for transformed hepatocytes [193]. The hedgehog pathway was found to be involved in progression of SH and development of fibrosis by inducing osteopontin [169]. Moreover, hedgehog/YAP signalling activates hepatic stellate cells in patients carrying the PNPLA3 I148M variant that is associated with risk of NASH progression [194,195], accompanied by metabolic changes, such as anaerobic glycolysis, favouring HCC development.

## 4. Discussion: Open Questions

We have presented the PTAS (priming, triggering, adaptation, senescence) model for SH that reflects our interpretation of the causal chain from aetiology to phenotype on the basis of experimental studies in correlation with observations in humans.

However, at present no single animal model can account for all elements of SH in humans with regard to aetiology and phenotype. One important future goal will therefore be the creation of an animal model that represents the PTAS mechanism whenever exposed to an aetiology that is relevant for human SH and exhibits the mature SH phenotype, including sequelae. This model will then serve as a strong support for the PTAS model and allow further studies on the development of SH.

**Figure 1:**
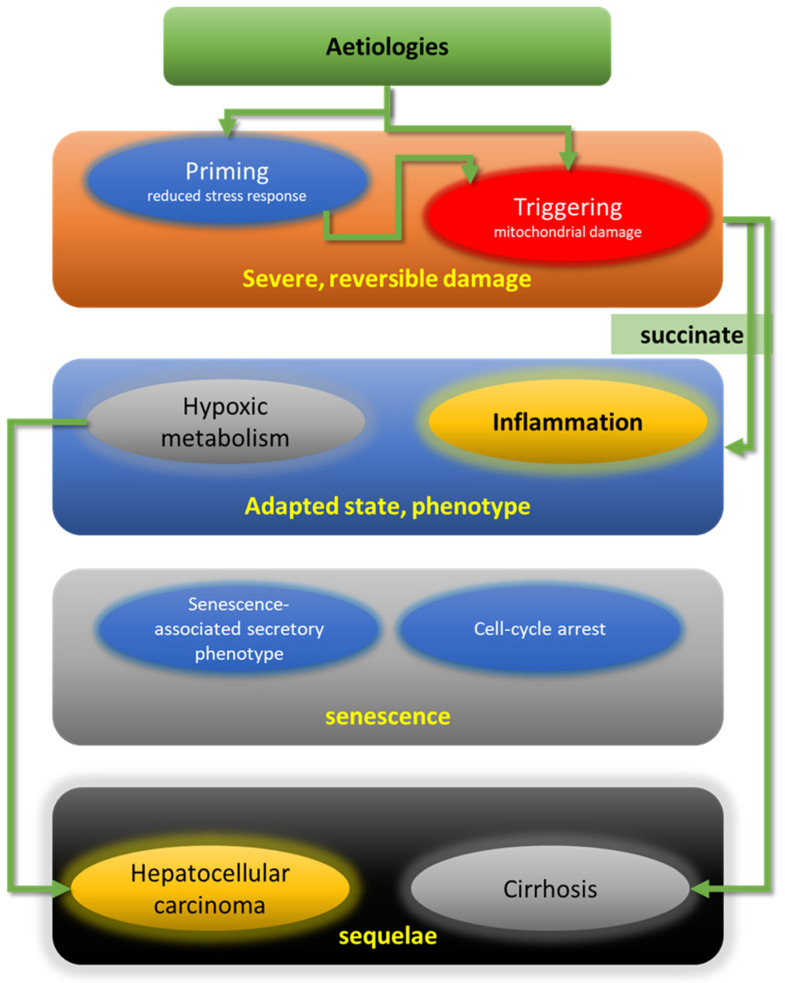
Overview on the PTAS model for steatohepatitis.

In Figure 1 the PTAS model is summarized: a two-pronged initial, acute insult leads to liver (hepatocyte) damage, by simultaneous or sequential priming and triggering. Neither of these alone can produce damage that is severe or long-lasting enough to lead to the CDM and eventually to the SH phenotype. We suppose that after priming and triggering, the liver damage may be pronounced but reversible (after cessation of priming and triggering events); however, after adaptation, which is essential for survival and sustained basic liver function of the hepatocyte under persisting conditions of priming and triggering, a new steady-state is reached. To avoid metabolic breakdown (caused by impaired function of the ETC and reduced ATP production) the cell adapts by switching to an alternative metabolism, similar to that in hypoxia, which is, on the one hand, less efficient and results in reduced hepatocyte function but, on the other, allows survival. Whereas acute hypoxia causes transcriptional arrest, during chronic hypoxia Hif-2 activates a translational program that enables anaerobic metabolism [131,142,143]—a process that is known as the ‘Warburg-effect’ and is also found in cancer cells. Increased succinate efflux triggers activation of stellate cells that leads to increased fibrosis/cirrhosis and inflammation through activated macrophages, both mediated by the SUCNR1 receptor. Hepatocellular pseudohypoxia may be regarded as a precancerous state that can lead to formation of HCC; however, the events during and after adaptation may also enter the senescence state as an alternative route, which prevents neoplastic transformation. The disadvantage of senescence is that through the SASP, hepatocytes persist in a metabolically minimally active state and can no longer contribute to normal liver function. Due to the regenerative capacity of the liver, this loss may be temporarily compensated by cell proliferation and increase of liver mass; however, liver failure may be the final consequence.

There are similarities of the mechanism described here with processes occurring during ischemia and reperfusion injury, with the difference that under these conditions, instead of a pseudohypoxic state, true hypoxic signalling develops; however, there is clear evidence that succinate acts as a danger signal also in this case.

Moreover, although evidence for adaptation and senescence is rather widespread in the literature, there are still gaps in establishing mechanistic links between late and early parts of the mechanism.

### 4.1. Open Questions Regarding Priming and Triggering Events

It was already mentioned above that in the literature the function of PPARα is almost exclusively related to lipid metabolism rather than stress response. There is, however, no doubt about the causal involvement of PPARα in human SH [61,63,64,82,83,118,196,197]. Since the causes for downregulation of PPARα and stress response are not fully elucidated, it is necessary to also revisit the metabolism-related approach of SH pathogenesis [18,31,86,198,199,200], in addition to more work on the proposed mechanism by activation of the AhR [67].

The role of mitochondria in triggering human SH is still insufficiently elucidated, although mitochondrial involvement in SH pathogenesis has been known for a long time [19,20,23,24,25,27,46,113,120]. The crucial factor of mitochondrial dysfunction in the PTAS model is that it is aggravated by impaired stress response and leads to disruption of oxidative phosphorylation and the tricarboxylic acid cycle, followed by succinate release and hypoxic signalling.

Hence, the effect of succinate, which is clearly involved in SH, and is known as part of a damage-associated molecular pattern, is another topic that merits more attention [40,56,106,107,136,201,202]. It is probably closely associated with the (pseudo-)hypoxic state that is proposed to play a role at the transition to adaptation [32,35,57,141,145,203,204]. Their causal connection, specifically in the context of SH, is still insufficiently studied both in human SH and in animal models. It is quite interesting that succinate may also link the metabolic state of adipose tissue to that of the liver [40], and future studies might be facilitated by a PTAS-relevant animal model.

### 4.2. Open Questions Regarding Adaptation

In the PTAS model, adaptation mitigates the damage incurred during priming and triggering. During adaptation the hepatocyte reaches a new metabolic state that is stable but different from that of the ‘naïve’ hepatocyte. It allows mitigation of the damage at the expense of functionality and reduced resilience towards further insults. It eventually leads to the formation of the SH phenotype and constitutes a transition state towards neoplastic development or senescence.

Adaptation is characterized by a (pseudo-)hypoxic state, and hypoxic signalling has been described for human SH in the literature [32,33,35,57,144,145,203,204,205,206,207,208]. Adaptation is the committed step of SH development; however, this stage is quite complex, e.g., it is made persistent through several feedback loops to priming [57,205] and triggering [117,209,210], and many of these processes overlap and make their analysis difficult. Therefore, in human SH direct evidence is scarce, linking, e.g., succinate to hypoxic signalling, or the consequences of pseudohypoxia on cell cycle arrest and senescence. During adaptation, hepatocytes communicate their status to other cell types, such as stellate cells, e.g., by release of succinate [40,56,101,106,136,211]. Here, too, a specific PTAS-aligned animal model would be helpful.

### 4.3. Open Questions Regarding Senescence

Besides the fully adapted state, senescence is the second ‘endpoint’ in the PTAS model. We think that fully developed SH consists of both adapted hepatocytes and senescent cells (hepatocytes, cholangiocytes, and stellate cells). While there is a substantial body of literature that indicates senescence as a cellular state in SH [36,37,38,39,177,178,179,180,181,212,213,214,215,216,217,218], there are scarcely any functional data about induction of senescence and the fate of senescent cells, the potential for spreading of senescence via the SASP and escape from senescence with the potential for neoplastic transformation.

## 5. Conclusions: What We Presently Know and Do Not Know, and Some Diagnostic and Therapeutic Options

We think that the PTAS mechanism described above represents a good ‘working’ model of SH since it accommodates a broad spectrum of experimental findings (morphology, metabolic alterations, senescence, and sequelae) in animal models and humans [16]. We have certainly not included all phenomena associated with SH, either because we think that they are a result and not part of PTAS, or because they accrued from models that do neither produce the phenotype nor have the aetiology in common with human SH [16]. The PTAS model is intended as a starting point and guideline for focused research on this complex disease mechanism.

### Diagnostic and Therapeutic Options

Armed with better understanding of the mechanistic underpinnings of the cascade that eventually leads to irreversible liver damage in SH, we provide a few ideas for diagnostic and therapeutic intervention. It should be noted, however, that besides the CDM, different initiating factors of different aetiologies also represent specific therapeutic targets [15].

A first diagnostic indicator may be serum succinate, which was also found elevated in perfusate of transplant livers that undergo ischemia reperfusion injury [219]; succinate may thus be a discriminant between steatosis and SH. It may also be of interest to use the expression profiles of peripheral blood mononuclear cells to detect the signature of hepatic (pseudo)hypoxic zones in the liver [145]. Finally, at later stages we may aim at detecting circulating senescence markers, such as soluble urokinase plasminogen activator receptor [218] or specific signatures in circulating extracellular vesicles [220,221].

The important therapeutic action at the transition from steatosis to SH will be to revert mitochondrial damage (e.g., by administration of mitochondrially targeted antioxidants/SDH uncouplers [222,223,224,225], which have already been used in clinical trials [46]), improve mitochondrial quality control [226], and prevent pseudohypoxia (e.g., by Hif-1 and Hif-2 inhibitors). Late-intervention therapy might be supported by senolytics [39,227].

There are still gaps in explaining the mechanism of MDB formation, the cause of ballooning, and similar morphological hallmarks. A functional role for keratins has been implied in this process [51,59,81,125], and apoptosis-triggered release of keratin 18 [228] and keratin 18-related tissue polypeptide-specific antigen into the circulation has been reported to be a diagnostic marker for SH [229]. However, with the aid of a framework model, it is possible that these gaps can be closed more rapidly and with greater focus.

## Figures and Tables

**Table 2:** Factors that trigger acute mitochondrial damage in the liver.

Factor	Mechanism	Model
Porphyrinogens (DDC, griseofulvin); liver toxins	Inhibition of ferrochelatase (haem deficiency)	Intoxication mouse models [28,50,51,52,53,100]; DILI [12,13,14]
Excess keratin 8 ^1^	Impaired mitochondrial QC via Pirh2	Keratin 18^−/−^ mouse model, HFD mouse model, human NASH [65,74,78,79,80,81]
HFD ^2^	Increased ROS production by β-oxidation of fatty acids	HFD model, human NASH [18,22,27,31,93,117]

^1^ Keratin 8 excess might prime due to impaired NADPH production in the TCA cycle, reducing mitochondrial GSH, and trigger through increased ROS production due to accumulating mitochondrial damage. ^2^ HFD might need a separate trigger since HFD mouse models do not produce the full SH phenotype [16].

**Table 3:** Outcome of priming and triggering, leading to the CDM.

Stage	Outcome	Effect
priming	Persistently reduced PPARα	pseudohypoxia
triggering	Damage to mitochondrial ETC Reduced mitochondrial QC succinate ↑	DAMP, ROS ↑, succinate ↑, pseudohypoxia, cell cycle arrest DAMP, stellate cell activation, immune response, inflammation
	Keratin 8 excess	Reduced mitochondrial QC, cell cycle arrest ^1^

^1^ The implication of Pirh2 and HuR in SH is presently not clear but represents a testable hypothesis.
